# Metabolomics and Transcriptomics Analyses of Curcumin Alleviation of Ochratoxin A-Induced Hepatotoxicity

**DOI:** 10.3390/ijms25010168

**Published:** 2023-12-21

**Authors:** Peng Hui, Xianrui Zheng, Jiao Dong, Fan Lu, Chao Xu, Huan Qu, Xiaoyang Zhu, Yoshinobu Uemoto, Xiaoyang Lv, Zongjun Yin, Wei Sun, Wenbin Bao, Haifei Wang

**Affiliations:** 1 Key Laboratory for Animal Genetics, Breeding, Reproduction and Molecular Design, College of Animal Science and Technology, Yangzhou University, Yangzhou 225009, China; 2 College of Animal Science and Technology, Anhui Agricultural University, Hefei 230036, China; 3 Graduate School of Agricultural Science, Tohoku University, Sendai 980-8572, Japan; 4 International Joint Research Laboratory in Universities of Jiangsu Province of China for Domestic Animal Germplasm Resources and Genetic Improvement, Yangzhou University, Yangzhou 225009, China

**Keywords:** Ochratoxin A, curcumin, hepatotoxicity, metabolome, transcriptome, pantothenic acid

## Abstract

Ochratoxin A (OTA) is one of the mycotoxins that poses a serious threat to human and animal health. Curcumin (CUR) is a major bioactive component of turmeric that provides multiple health benefits. CUR can reduce the toxicities induced by mycotoxins, but the underlying molecular mechanisms remain largely unknown. To explore the effects of CUR on OTA toxicity and identify the key regulators and metabolites involved in the biological processes, we performed metabolomic and transcriptomic analyses of livers from OTA-exposed mice. We found that CUR can alleviate the toxic effects of OTA on body growth and liver functions. In addition, CUR supplementation significantly affects the expressions of 1584 genes and 97 metabolites. Integrated analyses of transcriptomic and metabolomic data showed that the pathways including Arachidonic acid metabolism, Purine metabolism, and Cholesterol metabolism were significantly enriched. Pantothenic acid (PA) was identified as a key metabolite, the exogenous supplementation of which was observed to significantly alleviate the OTA-induced accumulation of reactive oxygen species and cell apoptosis. Further mechanistical analyses revealed that PA can downregulate the expression level of proapoptotic protein BAX, enhance the expression level of apoptosis inhibitory protein BCL2, and decrease the level of phosphorylated extracellular signal-regulated kinase 1/2 (pERK1/2). This study demonstrated that CUR can alleviate the adverse effects of OTA by influencing the transcriptomic and metabolomic profiles of livers, which may contribute to the application of CUR in food and feed products for the prevention of OTA toxicity.

## 1. Introduction

Ochratoxin A (OTA), produced by Aspergillus and Penicillium species, is one of the most common contaminants in feed and food, and its bioaccumulation through the food chain poses a serious threat to human and animal health [1]. OTA is absorbed into the blood from the gastrointestinal tract, bound to serum proteins, and transported to other tissues and organs through the blood, causing hepatotoxicity, nephrotoxicity, and immunotoxicity [2,3,4]. The accumulation and biotransformation of OTA occur predominantly in the liver and kidney, whereas the liver is the main organ for toxicological studies [1,5]. Studies have shown that OTA exposure can induce oxidative stress by reactive oxygen species (ROS) overproduction [6,7]. In addition, OTA can also reduce the antioxidant defense of cells by inhibiting the expression of AP-1 and Nrf2 proteins that participate in the regulation of the expression of glutathione, glutathione S-transferase, and other cytoprotective enzymes [8]. As one of the targets of OTA, the liver toxicity of OTA has gained much attention for the risk assessment and toxic prevention of this mycotoxin. However, the molecular mechanisms underlying the toxicological process of OTA in the liver remain poorly understood.

Curcumin (CUR) is a natural polyphenol compound extracted from the rhizomes of Curcuma longa and is widely used as a spice, traditional medicine, as well as a food and feed additive. As a natural plant extract, CUR has various pharmacological activities such as antioxidant, anti-inflammatory, antibacterial, and fungal activities [9,10]. The phenolic group of CUR is responsible for its ability to react with reactive species and may be one of the mechanisms by which CUR administration protects cells from oxidative damage. The use of natural plant active compounds has emerged as a potential approach to reduce the toxicity caused by environmental contaminants such as fungal poisoning. CUR has been found to attenuate OTA and Aflatoxin B1-induced hepatotoxicity and nephrotoxicity in rats, chickens, and ducks [9,10,11,12]. CUR can also protect human intestinal epithelial cells from H_2_O_2_-induced tight junction disruption through the heme oxygenase 1 pathway [13]. Meanwhile, CUR increased the expression of multiple detoxifying enzymes in the liver, small intestine, and kidney tissues of mice to protect against toxic substance-induced liver and kidney injury [14]. These reports indicated the important functions of CUR in reducing the toxic effects of OTA; however, the underlying mechanisms of the biological processes have not been fully explored.

In this study, to explore the changes in gene expression and metabolite abundance as well as the molecular mechanisms of CUR in alleviating OTA toxicity, we performed transcriptomics and metabolomics analyses of livers derived from OTA-exposed mice. We found that CUR can alleviate the OTA-induced hepatotoxicity and affect the expression of genes and metabolites involved in the toxicological process of OTA. Pantothenic acid (PA) was found as a key metabolite in attenuating the toxic effects of OTA on liver cells. The results of this study revealed the roles of CUR in alleviating the toxic effects of OTA and uncovered the underlying transcriptomic and metabolic patterns, which may facilitate the use of CUR as additives in food and feed for the prevention and control of OTA toxicity.

## 2. Results

### 2.1. CUR Attenuates OTA-Induced Hepatotoxicity in Mice

**Figure 1:**
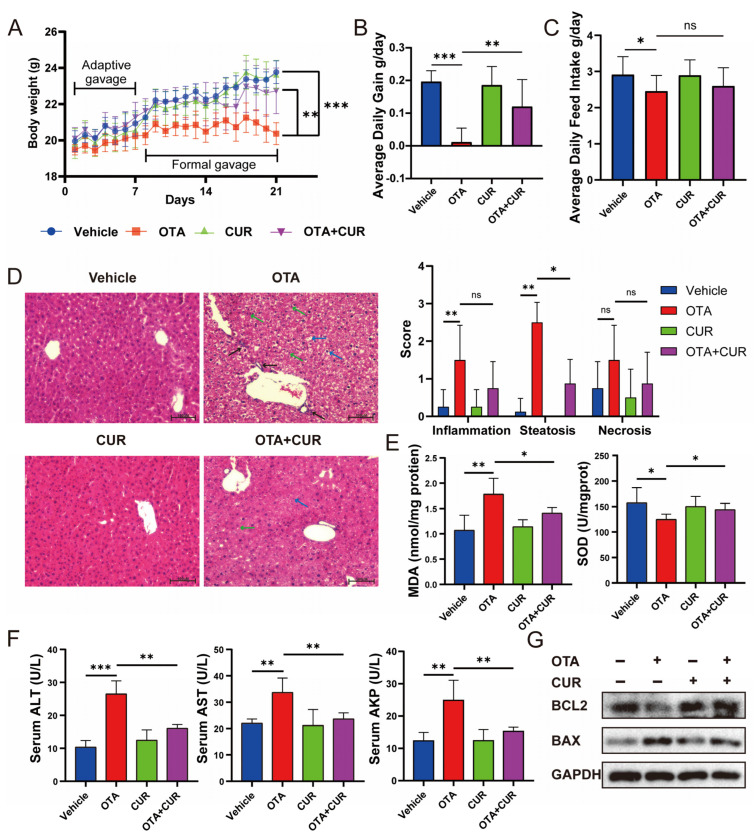
Effects of CUR supplementation on body weight, feed intake, and liver injury in OTA-exposed mice. (**A**) Body weight changes in mice from 1 to 21 days. (**B**) Average daily gain of mice. (**C**) Average daily feed intake of mice. (**D**) H&E-staining of mouse liver and severity scores of inflammation, steatosis, and necrosis for each group. Black arrows indicate mononuclear cell infiltration. Blue arrows indicate swollen cells. Green arrows indicate hepatic macrophages (Kupfer cells). The scale bar represents 100 μm. (**E**) Hepatic MDA and SOD levels in mice. (**F**) Serum levels of liver injury markers ALT, AST, and ALP in mice. (**G**) Expression levels of apoptotic proteins BCL2 and BAX in mice liver. Vehicle: 5 mL/kg b.w. corn oil; OTA: 0.5 mg/kg b.w. OTA; CUR: 100 mg/kg b.w. CUR. OTA+CUR: 0.5 mg/kg b.w. OTA+100 mg/kg b.w. CUR. Data are shown as the mean ± SD of three independent experiments (*n* = 3). * *p* < 0.05, ** *p* < 0.01, *** *p* < 0.001, ns: not significant.

We recorded changes in body weight and feed intake from day 1 to day 21 of the mice in each group. The results showed that OTA exposure resulted in significant (*p* < 0.01) decreases in body weight, average daily gain (Figure 1A,B), and average daily feed intake (Figure 1C) in mice. However, CUR addition significantly (*p* < 0.05) reduced the toxic effects caused by OTA exposure (Figure 1A–C). To explore whether CUR addition alleviates OTA-induced hepatotoxicity in vivo, we examined the histomorphological changes in the liver. The results showed that OTA exposure resulted in mononuclear cell infiltration and moderate to severe steatosis and necrosis, and CUR addition significantly alleviated the histological damage induced by OTA exposure (Figure 1D). The activity of key antioxidant enzymes was determined to further explore whether CUR could alleviate OTA-induced oxidative stress. The results revealed that the MDA content increased with OTA treatment but was markedly inhibited with CUR supplementation, and the SOD was reduced by the OTA, while CUR restored the reduction caused by the OTA (Figure 1E). We further examined the serum levels of biomarkers of liver injury including ALT, AST, and ALP. The results showed that OTA exposure significantly (*p* < 0.01) increased ALT, AST, and ALP activities in serum. In contrast, CUR addition obviously alleviated the effects of OTA on the activities of ALT, AST, and ALP (Figure 1F). At the same time, we detected the expression levels of apoptosis-related proteins BCL2 and BAX. The results showed that CUR considerably alleviated OTA-induced hepatocyte apoptosis (Figure 1G). Together, these results indicated that CUR can alleviate the OTA-induced hepatotoxicity in mice.

### 2.2. CUR Alleviates the Changes in Hepatic Transcriptomics Caused by OTA

**Figure 2:**
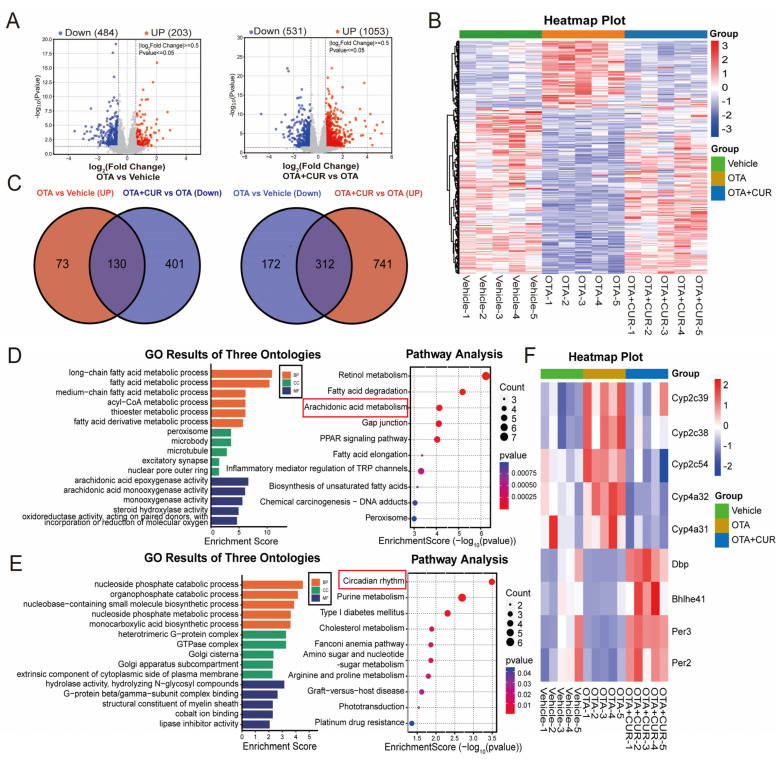
CUR restores hepatic transcriptomic changes induced by OTA exposure in mice. (**A**) Volcano plot of DEGs between different groups. (**B**) Heatmap of cluster analysis based on expression levels of DEGs. (**C**) Venn diagram of DEGs between different groups. (**D**) GO terms and KEGG pathways of DEGs upregulated in the OTA group and downregulated in the OTA+CUR group. (**E**) GO terms and KEGG pathways of DEGs downregulated in the OTA group and upregulated in the OTA+CUR group. (**F**) Heatmap of DEGs within Arachidonic acid metabolism and Circadian rhythm signaling pathways. Vehicle: 5 mL/kg b.w. corn oil; OTA: 0.5 mg/kg b.w. OTA; OTA+CUR: 0.5 mg/kg b.w. OTA+100 mg/kg b.w. CUR.

To explore the potential regulators of CUR associated with OTA-induced liver injury remission, we collected mouse liver samples for transcriptomic analysis using RNA-seq. This sequencing yielded a total of 168.67 G of raw data, and 162.59 G of clean data after quality control, with an average of 43.1 million clean reads per sample (Table S1). Differential expression analysis identified 687 DEGs, with 203 upregulated and 484 downregulated DEGs in the OTA group compared with the vehicle group (Figure 2A and Table S2). In addition, 1584 DEGs were identified, with 1053 upregulated and 531 downregulated DEGs in the OTA+CUR group compared to the OTA group (Figure 2A and Table S3). Hierarchical clustering analysis showed different patterns of gene expression among the three groups, whereas genes in the OTA+CUR group showed similar expression patterns with those in the vehicle group (Figure 2B).

To further explore the effects of CUR on gene expression, we intersected the DEGs in the OTA group and the OTA+CUR group, and found 130 DEGs upregulated in the OTA group and downregulated in the OTA+CUR group as well as 312 downregulated in the OTA group and upregulated in the OTA+CUR group (Figure 2C and Table S4). We then performed functional enrichment analysis for the intersected DEGs and found that upregulated DEGs in the OTA group were mainly enriched in biological processes including the fatty acid metabolic process and acyl-CoA metabolic process, and pathways including Retinal metabolism and Arachidonic acid metabolism (Figure 2D and Table S5). The downregulated DEGs in the OTA group were mainly enriched in biological processes including the nucleoside phosphate catabolic process and organophosphate catabolic process, and pathways including the Circadian rhythm and Purine metabolism (Figure 2E and Table S6).

The DEGs involved in the Arachidonic acid metabolism and Circadian rhythm signaling pathway played regulatory roles in mycotoxin toxic effects [15,16]. A gene expression heat map showed that the expression of the genes involved in Arachidonic acid metabolism were significantly upregulated in the OTA group but downregulated in the OTA+CUR group, while the expression of the genes involved in the Circadian rhythm signaling pathway demonstrated a contrary trend (Figure 2F). These results indicated the potential roles of these genes in the alleviation of OTA-induced hepatotoxicity by CUR.

### 2.3. CUR Improves the Changes in Liver Metabolomics Caused by OTA

**Figure 3:**
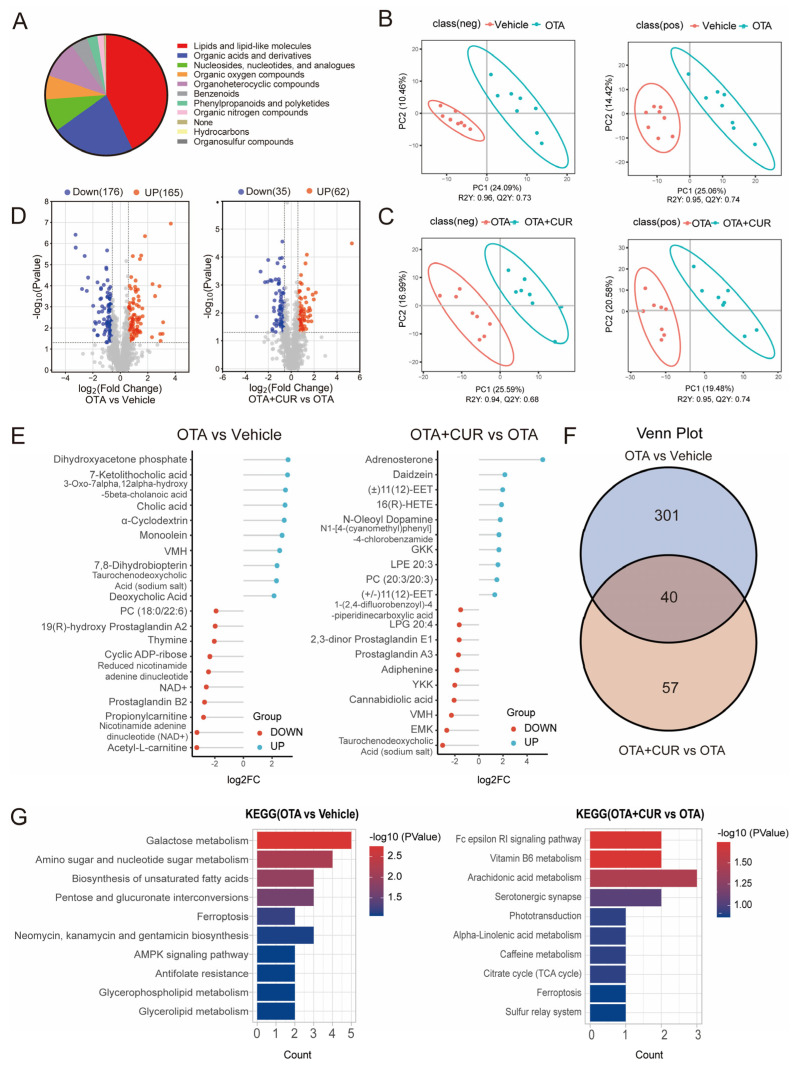
CUR alleviates OTA-induced hepatic metabolic disorders in mice. (**A**) Classification of the identified metabolites. (**B**) OPLS-DA for differential metabolites between vehicle and OTA in positive and negative ion mode. (**C**) OPLS-DA for differential metabolites between OTA and OTA+CUR in positive and negative ion mode. (**D**) Volcano plot of differential metabolites between different groups. (**E**) Top 20 differential metabolites between different groups. (**F**) Venn diagram of differential metabolites between different groups. (**G**) KEGG pathways enriched by differential metabolites. Vehicle: 5 mL/kg b.w. corn oil; OTA: 0.5 mg/kg b.w. OTA; OTA+CUR: 0.5 mg/kg b.w. OTA+100 mg/kg b.w. CUR.

To assess the effect of CUR on OTA-induced metabolic alterations, we performed untargeted metabolomics of the liver using LC-MS/MS. A classification of the metabolites showed that all metabolites mainly belonged to 10 categories, including Lipids and lipid-like molecules, Organic acids, compounds, and Organoheterocyclic derivatives (Figure 3A). Orthogonal partial least squares discriminant analysis (OPLS-DA) showed obvious metabolomic alterations among the three groups (Figure 3B,C). Compared to the vehicle group, 341 significantly differential metabolites were detected in the OTA group, including 176 downregulated and 165 upregulated metabolites (Figure 3D and Table S7). Furthermore, 97 differential metabolites were detected in the OTA+CUR group compared to the OTA group, including 35 downregulated and 62 upregulated metabolites (Figure 3D and Table S8). The top 10 differentially upregulated and downregulated metabolites were shown in Figure 3E. Integrative analysis indicated that more than 41% of the differential metabolites were rescued by CUR treatment compared with the OTA group (Figure 3F). We then performed KEGG enrichment analysis for the differential metabolites. The results showed that the differential metabolites induced by OTA exposure were significantly enriched in pathways including Galactose metabolism, Amino sugar and nucleotide sugar metabolism, and the Biosynthesis of unsaturated fatty acids (Figure 3F and Table S9). However, the differential metabolites in the OTA+CUR group were mainly enriched in the pathways including the Fc epsilon RI signaling pathway, Vitamin B6 metabolism, and Arachidonic acid metabolism (Figure 3G and Table S10).

### 2.4. Integrated Analyses of Metabolomics and Transcriptomics Data

**Figure 4:**
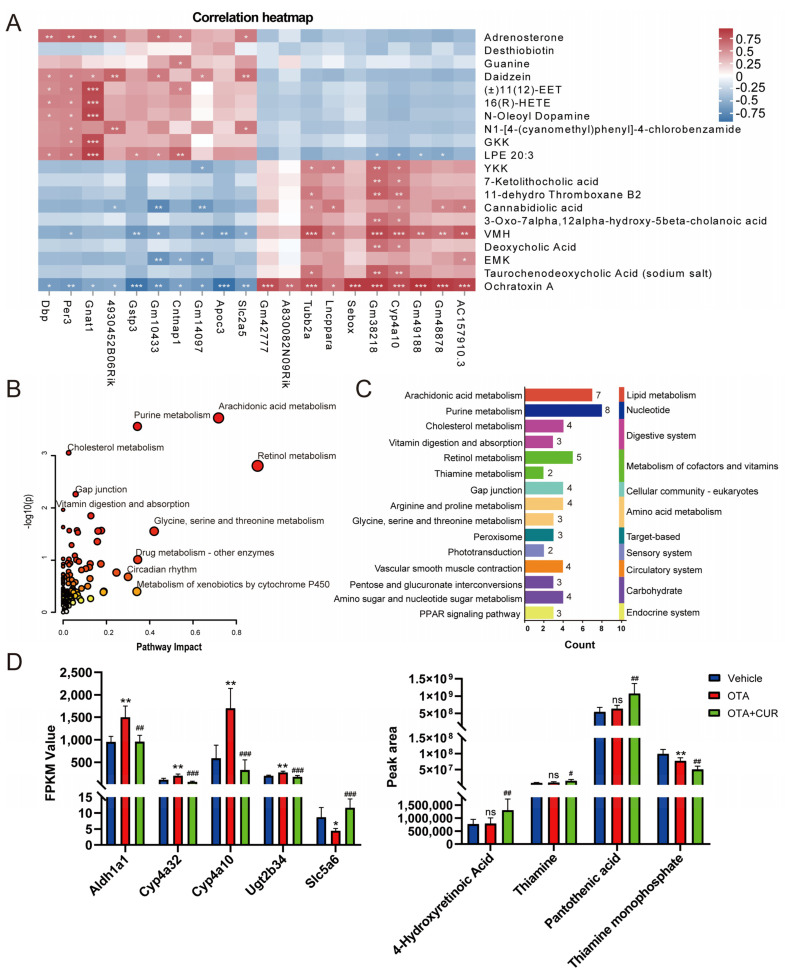
Integrated analysis of differential metabolites and DEGs. (**A**) Correlations between the top 20 DEGs and differential metabolites. (**B**) KEGG pathways enriched by DEGs and differential metabolites. (**C**) Secondary classification for the significantly enriched pathways. (**D**) Expression levels of DEGs and differential metabolites in the Retinol metabolism, Thiamine metabolism, and Vitamin digestion and absorption pathways. * *p* < 0.05, ** *p* < 0.01, *** *p* < 0.001, ns: not significant (compared to Vehicle group). ^#^ *p* < 0.05, ^##^ *p* < 0.01, ^###^ *p* < 0.001 (compared to OTA group).

To identify the key metabolites and genes involved in CUR alleviating OTA-induced toxicity, we performed a combined analysis of differentially expressed metabolites and genes in the OTA+CUR group and the OTA group. The top 20 differential metabolites and their association with DEGs are shown in (Figure 4A and Table S11). KEGG enrichment analysis for DEGs and differential metabolites showed that these metabolites and genes were significantly enriched in pathways such as Arachidonic acid metabolism, Purine metabolism, Cholesterol metabolism, and Retinol metabolism (Figure 4B and Table S12). The secondary classification of these pathways indicated that the metabolic pathways related to vitamin metabolism including Retinol metabolism, Thiamine metabolism, and Vitamin digestion and absorption were significantly enriched (Figure 4C). The genes (*Aldh1a1*, *Cyp4a32*, *Cyp4a10*, and *Ugt2b34*) in the Retinol metabolism pathway were significantly upregulated, and the *Slc5a6* gene in the vitamin digestion and absorption pathway was significantly downregulated after OTA exposure, whereas CUR addition significantly reversed the changes in the expression of these genes (Figure 4D). The differential metabolites including Pantothenic acid (PA), 4-Hydroxyretinoic Acid, and Thiamine did not significantly change after OTA exposure. However, their expression levels were obviously upregulated when CUR was supplemented, with Pantothenic acid (PA) being the most significant (*p* < 0.01).

### 2.5. PA Attenuates OTA-Induced Hepatotoxicity

**Figure 5:**
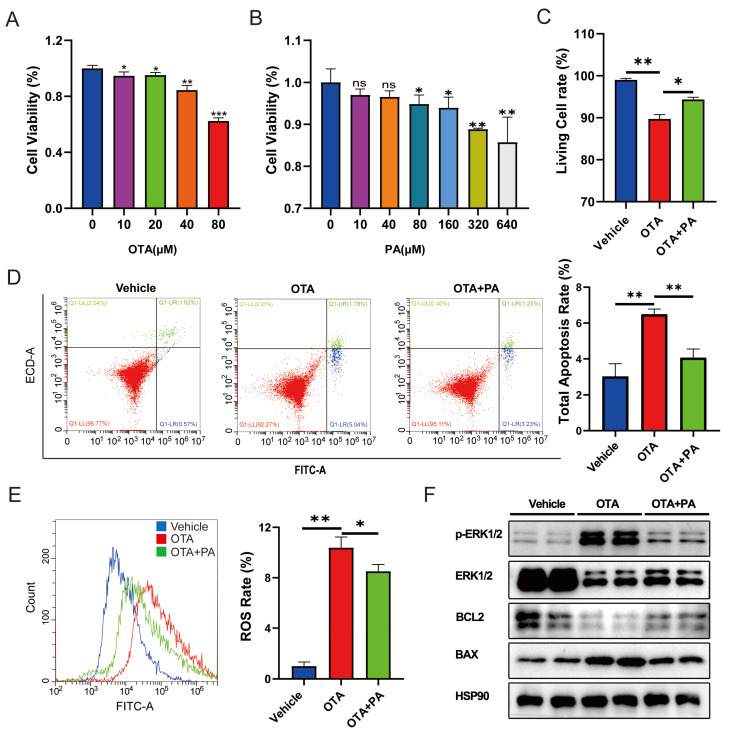
Effects of PA on OTA-induced hepatotoxicity. (**A**,**B**) Effects of different concentrations of OTA and PA on the cell viabilities. (**C**) Effects of PA on OTA-induced cell death. (**D**) Effects of PA on OTA-induced apoptosis. (**E**) Effect of PA on OTA-induced ROS levels in AML-12 cells. (**F**) Effects of PA on OTA-induced expressions of apoptosis-related proteins in AML-12 cells. Vehicle: cells without OTA exposure; OTA: cells with ZEA exposure; OTA+PA: cells with 80 μM OTA exposure and 40 μM PA addition. Data are shown as the mean ± SD of three independent experiments (*n* = 3). * *p* < 0.05, ** *p* < 0.01, *** *p* < 0.001, ns: not significant.

PA plays central roles in the crossover between amino acid catabolism, glycolysis, and fatty acid metabolism [17]. To verify the potential effect of PA on OTA-induced hepatotoxicity, we analyzed the ROS and cell apoptosis levels in AML-12 cells exposed to OTA with exogenous PA addition. First, we measured the cell viability of cells exposed to different concentrations of OTA and observed that OTA reduced the cell viability in a dose-dependent manner (Figure 5A) and 80 μM of OTA was used in subsequent studies. In parallel, we examined the effects of different concentrations of PA on cell viability and finally selected 40 μM PA for subsequent experiments (Figure 5B). We further investigated the potential effects of exogenous PA supplementation on OTA-induced hepatotoxicity. We observed that PA supplementation significantly (*p* < 0.05) alleviated OTA-induced cell viability inhibition (Figure 5C). In addition, PA supplementation dramatically alleviated OTA-induced cell apoptosis (Figure 5D) and ROS (Figure 5E). Western blot showed that compared to the cells exposed to OTA, PA addition downregulated the expression level of proapoptotic protein BAX and enhanced the expression level of apoptosis inhibitory protein BCL2 (Figure 5F). Furthermore, PA addition dramatically decreased the level of phosphorylated extracellular signal-regulated kinase 1/2 (pERK1/2) compared with that of the OTA group (Figure 5F).

## 3. Discussion

Integrated analyses of transcriptome and metabolome enable the identification of the important metabolites and regulators involved in the complex biological processes [18,19]. Herein, we observed that OTA exposure significantly altered the transcriptomic and metabolic profiles of the liver, indicating the associations of the affected genes and metabolites with OTA toxicity. Furthermore, CUR can obviously reverse the changes in the expression of the genes and metabolites involved in OTA hepatotoxicity, which suggested that CUR reduces the adverse effects of OTA by reestablishing the gene expression and metabolite expression patterns. Meanwhile, we found that the Arachidonic acid metabolism pathway was significantly enriched in the OTA-treated group, suggesting that it may play an important role during OTA-induced hepatotoxicity. Vitamin metabolism-related pathways such as Retinol metabolism, Thiamine metabolism, and Vitamin digestion and absorption were significantly enriched in the OTA+CUR group. In addition, we identified PA as a critical metabolite potentially involved in CUR attenuating OTA-induced hepatotoxicity. The results revealed the important metabolic pathways and regulators involved in the processes of CUR alleviating OTA toxicity, which provided new insights into the molecular mechanisms of the biological processes.

In this study, we found that the upregulated DEGs induced by OTA exposure were significantly enriched in Arachidonic acid metabolism. Arachidonic acid can be converted to various metabolites through the cytochrome P450 enzyme system [20]. Cytochrome P450 family members are crucial early responsive genes that catalyze the metabolism of mycotoxins [21]. CYP450 activation is thought to be a significant signal for the metabolic activation of toxic or carcinogenic metabolites [22]. In addition, the overexpression of CYP450 is positively correlated with the morphological and functional impairment caused by oxidative stress [23]. It has been reported that CYP450 enzymes are significantly upregulated following AFB1 exposure and then participate in AFB1-induced duodenal injury [24]. Herein, we found that cytochrome P450 family members *Cyp2c39*, *Cyp2c38*, *Cyp2c54*, *Cyp4a32*, and *Cyp4a31* were significantly upregulated after OTA exposure, while their expressions were significantly downregulated when CUR was added. These results indicated that CUR may alleviate the OTA toxicity by affecting the expression of cytochrome P450 genes.

Furthermore, we observed the obvious down-expression of genes (*Dbp*, *Bhlhe41*, *Per3*, and *Per2*) enriched in the Circadian rhythm upon OTA exposure. Circadian rhythm disorders resulted in oxidative stress and increased intestinal and hepatic inflammation [25]. Therefore, the downregulated expression of the genes related to the Circadian rhythm may be one of the routes that OTA induces hepatotoxicity. It is evidenced that the overexpression of the core component of the Circadian rhythm Bmal1 obviously attenuates mycotoxin-induced liver inflammation and liver injury [16]. Interestingly, the downregulated DEGs (*Dbp*, *Bhlhe41*, *Per3*, and *Per2*) in the OTA group were significantly upregulated in the OTA+CUR group. CUR has been shown to reestablish circadian gene expressions and restore the locomotion ability in the Drosophila model of Huntington’s disease [26]. These findings suggested that CUR alleviated the hepatotoxicity of OTA by modulating the expression circadian genes (*Dbp*, *Bhlhe41*, *Per3*, and *Per2*).

Metabolomic analysis showed that OTA exposure resulted in the significant upregulation of Cholic acid and its metabolites including 7-Ketolithocholic acid, 3-Oxo-7alpha, and 12alpha-hydroxy-5beta-cholanoic acid. It is known that Cholic acid metabolism abnormalities are closely associated with liver disease, and changes in the cholic acid composition in the liver can promote inflammation, oxidative stress, and cell necrosis [27]. In addition, the dietary supplementation of Cholic acid resulted in substantial increases in liver necrotic lesions, multiple necrotic nuclei, abnormal hepatic triplet structures, and liver enzymes (ALT and AST) in mice [28,29]. In the OTA+CUR group, the levels of Cholic acid and its metabolites were significantly lowered. Thus, OTA may induce hepatotoxicity by disturbing Cholic acid metabolism, and CUR alleviated OTA toxicity by restoring the expression of the metabolites involved in Cholic acid metabolic processes.

Our integrative analyses of transcriptomics and metabolomics indicated that CUR addition significantly affected the levels of metabolites enriched in vitamin metabolism-related pathways, with PA being significantly upregulated in the OTA+CUR group. PA were mainly synthesized by probiotics that commonly colonize the colon [30,31]. CUR and its metabolites have been shown to influence the microbiota [32], which may promote PA production by regulating the abundance of intestinal microbes. Also, *Slc5a6*, a gene encoding the Sodium-dependent multivitamin transporter (SMVT), was significantly upregulated in the OTA+CUR group. SMVT is a transporter involved in the transport of biotin, pantothenic acid, and lipoic acid [33]. Therefore, we speculated that CUR could enhance PA uptake in hepatocytes via the SMVT transport system. However, PA was not significantly different in the OTA group, and we speculate that CUR can increase PA levels independently of OTA and benefit overall hepatocellular health. PA exerts antioxidant function and has protective effects during inflammation in the early stage of atherosclerosis [34]. In this study, we found that PA addition alleviated OTA-induced ROS production and cell apoptosis. Further mechanistic analysis showed that PA significantly decreased OTA-induced ERK1/2 phosphorylation, indicating that PA alleviated OTA-induced apoptosis in hepatic cells by inhibiting ERK1/2 phosphorylation. These findings provide novel insights into the mechanisms of CUR alleviating OTA-induced hepatotoxicity by affecting PA metabolic processes.

## 4. Materials and Methods

### 4.1. Experimental Animals

Thirty-two 5-week-old (19~21 g) BALB/c male mice were used in this study and purchased from Yangzhou University Laboratory Animal Center (Yangzhou, China). After 3 days of adaptive feeding, they were randomly divided into 4 groups (8 mice in each group): vehicle group (5 mL/kg b.w. corn oil), OTA group (0.5 mg/kg b.w. OTA), CUR group (100 mg/kg b.w. CUR), and OTA+CUR group (0.5 mg/kg b.w. OTA+100 mg/kg b.w. CUR). OTA (Pribolab, Qingdao, China) and CUR (MedChemExpress, Monmouth Junction, NJ, USA) were dissolved in corn oil. Experimental mice were housed in cages under standard conditions (temperature 25 ± 2 °C, 12-h day/night cycle) and provided with fresh, clean chow and water ad libitum. After one week of adaptive gavage, a 14-day formal gavage was conducted on all animals. The body weight of each animal was recorded daily. Animals in each treatment group were sacrificed by cardiac blood sampling after anesthesia. The liver was rapidly removed and frozen in liquid nitrogen for further use. In addition, a small piece of the liver was fixed in 4% formaldehyde solution for histopathological observation.

### 4.2. Histopathological Analysis

Fresh liver tissues were collected from the medial aspect of the left lobe of the mouse liver, washed with normal saline, and fixed in 4% paraformaldehyde for 24 h. Formaldehyde-fixed tissues were removed and trimmed into regular shapes and embedded in paraffin after stepwise dehydration. Embedded paraffin blocks were sliced into 5 μm slices using a microtome and dried at 37 °C for 72 h before deparaffinization. Hematoxylin-eosin staining was subsequently performed and histopathological examination was performed under a microscope. Liver lesions were scored using a defined scoring system [6] from three aspects: inflammation, steatosis, and necrosis by evaluating at least eight microscopic fields at 20× magnification.

### 4.3. Determination of Serum Hepatic Function Biomarkers

Serum alanine aminotransferase (ALT), aspartate aminotransferase (AST), and alkaline phosphatase (ALP) levels were measured using the testing kits according to the manufacturer’s instructions (Jiancheng Bioengineering Institute, Nanjing, China). Absorbance was measured at 510 nm using a Tecan Infinite 200 microplate reader (Tecan, Männedorf, Switzerland).

### 4.4. Detection of Oxidative Stress Markers

Collected mouse livers were subjected to tissue homogenization, and after centrifugation (3500 rpm, 20 min), the concentration of supernatants was detected using a BCA protein assay kit (Beyotime, Nantong, China). The contents of MDA and SOD in the supernatant of the mouse liver homogenate were then determined by the detection kit (Jiancheng Bioengineering Institute, Nanjing, China).

### 4.5. Metabolomics Analysis

In this study, untargeted metabolomics was performed based on liquid chromatography-mass spectrometry (LC-MS) technology. First, metabolites were extracted from the mouse liver samples and detected by the LC-MS/MS platform [18]. Raw data from mass spectrometry detection were preprocessed using Compound Discoverer 3.1 software. A qualitative analysis of the metabolites was also performed by combining the mzCloud, mzVault, and Masslist databases. Next, multivariate statistical analysis of metabolites, including partial least squares discriminant analysis (PLS-DA) hierarchical clustering and metabolite correlation analysis, was performed to reveal the differences in metabolic patterns among different groups and their relationships.

### 4.6. Transcriptomics Analysis

The total RNA from the liver tissues was extracted using Trizol reagent (Takara, Dalian, China), and mRNA was enriched with magnetic beads with Oligo (dT). Subsequently, a fragmentation buffer was added to break mRNA into short fragments, and double-stranded cDNA was synthesized. Double-stranded cDNA was purified using AMPure XP beads followed by end-repair, the addition of an A-tail, and the ligation of sequencing adapters. Finally, PCR amplification was performed, and PCR products were purified with AMPure XP beads to obtain the final sequencing library. Double-end sequencing was performed on an Illumina Hiseq-PE150 high-throughput sequencing platform. The expression of each gene was calculated using TopHat2 [35]. FPKM (fragments per kilobase of transcript per million mapped fragments) was computed using Stringtie [36]. Differentially expressed genes (DEGs) were screened using DESeq.2, and genes with |log2-fold change| ≥ 0.5 and a corrected *p*-value < 0.05 were considered as DEGs [37].

### 4.7. Integrated Analysis of Metabolomic and Transcriptomic Data

Pearson correlation analysis was conducted for differentially expressed genes and metabolites. DEGs and metabolites were submitted simultaneously to MetaboAnalyst 5.0 (https://www.metaboanalyst.ca/ (accessed on 13 July 2023)) for combined enrichment analysis. The significantly enriched pathways were subjected to biological annotation and secondary classification according to the KEGG pathway database (https://www.genome.jp/kegg/ (accessed on 14 July 2023)).

### 4.8. Cell Viability Assay

Mouse hepatocytes (AML-12) were seeded at a density of 1.5 × 10^4^ cells/mL in 96-well plates and placed in an incubator at 37 °C and 5% CO_2_ overnight. Different concentrations of OTA (20 μΜ, 40 μM, and 80 μM) and PA (10 μM, 40 μM, 80 μM, 160 μM, 320 μM, and 640 μM) were added to 96-well plates for 48 h at 37 °C and 5% CO_2_. Cell viability was analyzed using Cell Counting Kit-8 kit according to the manufacturer’s instructions (YEASEN, Shanghai, China). Absorbance was measured at 450 nm using a Tecan Infinite 200 microplate reader (Tecan, Switzerland).

### 4.9. Flow Cytometry

AML-12 cells at a 1 × 10^5^ cells/mL density were seeded in 6-well cell culture plates and placed in an incubator at 37 °C and 5% CO_2_ overnight. Cells were treated with OTA and PA (MedChemExpress, NJ, USA) for 48 h. Cells were washed once with PBS, digested with trypsin, and centrifuged at 1000 r/min for 5 min to collect cell samples. The ROS assay kit and Annexin V-FITC Apoptosis Detection Kit (Solarbio, Beijing, China) were used to quantify ROS and cell apoptosis levels by flow cytometry.

### 4.10. Western Blot

Total protein was extracted using RIPA lysate (Beyotime, Nantong, China) containing protease inhibitors and phosphatase inhibitors (CWBIO, Taizhou, China), and protein concentration was determined by the BCA protein assay kit (Beyotime, Nantong, China). Proteins were denatured in 5 × SDS-PAGE sample loading buffer (YEASEN, Shanghai, China), electrophoretically separated, and transferred onto PVDF membranes. After blocking in 5% skimmed milk for 2 h at room temperature, the proteins were incubated overnight at 4 °C with corresponding primary antibodies, including HSP90 (Proteintech, Wuhan, China), phospho-ERK1/2 (Cell Signaling Technology, Boston, MA, USA), ERK1/2 (Cell Signaling Technology, Boston, MA, USA), BCL2 (ABclonal, Wuhan, China), and BAX (ABclonal, Wuhan, China). Finally, the membranes were incubated in a horseradish peroxidase-labeled secondary antibody (CWBIO, Taizhou, China) for 2 h at room temperature. Protein expression was detected on a gel documentation system (Tanon, Shanghai, China) using ECL chemiluminescent chromogen solution (ABBKINE, Redlands, CA, USA).

### 4.11. Statistical Analysis

One-way ANOVA was performed using GraphPad Prism 8 software. The data were presented as mean ± standard derivation (SD). Statistical significance is shown as follows: * *p* < 0.05, ** *p* < 0.01, *** *p* < 0.001.

## 5. Conclusions

In conclusion, our findings show that CUR can effectively alleviate OTA-induced hepatotoxicity and provide global insights into the molecular mechanisms underlying the protective effects of CUR at the metabolomic and transcriptomic levels. In addition, PA was identified as a key metabolite involved in alleviating OTA toxicity by regulating oxidative stress and cell apoptosis. CUR may alleviate OTA-induced liver injury by modulating Arachidonic acid metabolism, the Circadian rhythm, and Vitamin B metabolism. Further functional validation demonstrated that the key metabolite PA alleviated OTA-induced hepatotoxicity by regulating oxidative stress and cell apoptosis. These findings extend our understanding of the key metabolites, genes, and metabolic processes associated with OTA exposure and reveal the potential metabolic and molecular targets of CUR to alleviate OTA-induced hepatotoxicity, providing a theoretical basis for the application of CUR in the prevention and control of OTA toxicity in animals.

## Data Availability

Data are contained within the article and Supplementary Materials.

## Supplementary Materials

The supporting information can be downloaded at: https://www.mdpi.com/article/10.3390/ijms25010168/s1.
